# Recombinant TAT-BMI-1 fusion protein induces ex vivo expansion of human umbilical cord blood-derived hematopoietic stem cells

**DOI:** 10.18632/oncotarget.15156

**Published:** 2017-02-07

**Authors:** Bruna Codispoti, Nicola Rinaldo, Emanuela Chiarella, Michela Lupia, Cristina Barbara Spoleti, Maria Grazia Marafioti, Annamaria Aloisio, Stefania Scicchitano, Marco Giordano, Giovanna Nappo, Valeria Lucchino, Malcolm A.S. Moore, Pengbo Zhou, Maria Mesuraca, Heather Mandy Bond, Giovanni Morrone

**Affiliations:** ^1^ Laboratory of Molecular Haematopoiesis and Stem Cell Biology, University Magna Græcia, Catanzaro, Italy; ^2^ Molecular Medicine Program, European Institute of Oncology, Milano, Italy; ^3^ Biogem S.C.a R.L., Ariano Irpino, Italy; ^4^ YCR Cancer Research Unit-Department of Biology, University of York, York, United Kingdom; ^5^ Department of Cell Biology, Memorial Sloan-Kettering Cancer Center, New York, NY, USA; ^6^ Department of Pathology and Laboratory Medicine, Weill Cornell Medical College, New York, NY, USA

**Keywords:** hematopoietic stem cells (HSCs), ex vivo expansion, BMI-1, TAT-fusion protein, protein transduction

## Abstract

Transplantation of hematopoietic stem cells (HSCs) is a well-established therapeutic approach for numerous disorders. HSCs are typically derived from bone marrow or peripheral blood after cytokine-induced mobilization. Umbilical cord blood (CB) represents an appealing alternative HSC source, but the small amounts of the individual CB units have limited its applications. The availability of strategies for safe *ex vivo* expansion of CB-derived HSCs (CB-HSCs) may allow to extend the use of these cells in adult patients and to avoid the risk of insufficient engraftment or delayed hematopoietic recovery.

Here we describe a system for the *ex vivo* expansion of CB-HSCs based on their transient exposure to a recombinant TAT-BMI-1 chimeric protein. BMI-1 belongs to the Polycomb family of epigenetic modifiers and is recognized as a central regulator of HSC self-renewal. Recombinant TAT-BMI-1 produced in bacteria was able to enter the target cells via the HIV TAT-derived protein transduction peptide covalently attached to BMI-1, and conserved its biological activity. Treatment of CB-CD34^+^ cells for 3 days with repeated addition of 10 nM purified TAT-BMI-1 significantly enhanced total cell expansion as well as that of primitive hematopoietic progenitors in culture. Importantly, TAT-BMI-1-treated CB-CD34^+^ cells displayed a consistently higher rate of multi-lineage long-term repopulating activity in primary and secondary xenotransplants in immunocompromised mice. Thus, recombinant TAT-BMI-1 may represent a novel, effective reagent for *ex vivo* expansion of CB-HSC for therapeutic purposes.

## INTRODUCTION

Hematopoietic stem cells (HSCs) are unquestionably the best-characterized somatic stem cells, and their transplant is routinely employed in clinical practice. Since its inception in the 1950s, hematopoietic stem cell transplantation (HSCT) has become established as a standard of care for a multitude of disorders, of both malignant and non-malignant nature, mostly affecting the hematopoietic system. Well over one million HSCTs have been performed thus far [1], with nearly 70,000 procedures being currently carried out annually worldwide [2]. Bone marrow (BM) initially represented the sole source of HSCs for transplantation, but in more recent years umbilical cord blood (CB), as well as peripheral blood of donors treated with cytokines that induce egress of HSCs from the bone marrow (mPB), have been recognized as convenient alternative sources of stem cells [3, 4]. In this regard, cord blood is particularly attractive owing to the availability of massive collections of cryopreserved samples and to the relative abundance of stem cells with extensive self-renewal potential. In addition, probably due to the immaturity of CB T lymphocytes, cord blood transplant is associated to a lower incidence of chronic graft-versus-host disease [5]. However, limited sample size, and consequently low absolute numbers of hemopoietic stem and progenitor cells present in individual cord blood units, have so far limited the use of CB for transplantation mostly to patients of pediatric age. Transplant of two partially HLA-matched cord blood units was introduced for adult patients for which no single units of adequate size were available [6, 7]. While improvements of engraftment and longer overall and leukemia-free survival were reported [8], only one of the two units is typically observed to provide long-term hematopoiesis (single unit dominance).

To circumvent the problems linked to the low cell doses, a variety of approaches have been attempted to achieve reliable *ex vivo* HSC expansion and/or to enhance their homing, and hence their engraftment upon transplantation (reviewed in [9]). Pioneering studies from Broxmeyer et al. [10], Piacibello et al. [11, 12] and several other groups defined combinations of hemopoietins that yielded robust expansion of CB-derived CD34^+^ cells in culture, however modest results were achieved when CB-HSCs expanded with hemopoietins alone were transplanted in pre-clinical assays [13], thus prompting the search for additional factors that could ensure a more efficient HSC amplification and engraftment.

A clinical trial in which one of the two CB units to be transplanted had been subjected to co-culture with allogeneic mesenchymal stromal cells demonstrated a strong expansion of CD34^+^ cells in the unit that had undergone co-culture. This resulted in a more rapid reconstitution of leukocyte populations in recipients, however long-term hematopoiesis was sustained predominantly by the non-expanded unit [14].

Several reports indicated that activation of Notch signaling results in accumulation of primitive hematopoietic progenitors in culture [15–19]. Based on this evidence, an *ex vivo* expansion approach was designed whereby CB-CD34^+^ cells were cultured for 16 days in the presence of an engineered form of the Notch ligand, Delta1 (Delta1^ext-IgG^), immobilized on the culture surface [20, 21]. This treatment resulted in a remarkable expansion of CD34^+^ cells, as well as a significantly accelerated myeloid recovery following transplant, but also in this case long-term reconstitution was supported by the non-expanded unit in most patients [20, 21].

The transient hematopoietic reconstitution observed in these trials may not necessarily reflect loss of long-term repopulating potential by expanded HSCs: single unit dominance has been linked to rejection, mediated by IFN-γ–secreting CD8^+^ T-cells, of the non-engrafting unit [22]. Therefore, if *ex vivo* expanded, T-cell-depleted CD34^+^ cells are co-transplanted with a non-manipulated CB unit, they may be eliminated through the activity of alloreactive T cells contained in the latter. In fact, a set of clinical trials in which T-cells from the manipulated cord blood unit were either not removed or infused together with expanded CD34^+^ cells, showed not only more rapid myeloid recovery but also persistent engraftment of the *ex vivo* treated HSCs. These trials regarded:

a recently-identified small molecule, termed StemRegenin-1 (SR-1), characterized as an aryl-hydrocarbon receptor agonist, which has been proven to induce striking expansion of CB-HSCs in culture [23]. In a Phase I/II trial, treatment with early-acting hemopoietins and SR-1 resulted in an over 330-fold increase of the CD34^+^ cell fraction, and 11 of 17 patients that received the amplified HSCs together with untreated CD34^-^ showed a predominant engraftment of these cells as well as a faster hematopoietic reconstitution [24];

treatment of isolated CB-CD133^+^ cells with hemopoietins and nicotinamide, an inhibitor of the Sirt1 deacetylase known to prevent HSC differentiation and promote their expansion in culture [25] as well as their homing. Co-transplantation of treated CD133^+^ cells and uncultured CD133^-^ cell fractions resulted in rapid neutrophil recovery and long-term engraftment of the expanded unit in 8 of 11 patients [26];

two protocols based on brief exposure of one whole CB unit to either dimethyl-prostaglandin E_2_ (dmPGE_2_) [27] or fucosyltransferase-VI and guanosine diphosphate fucose [28]. Both trials demonstrated accelerated myeloid reconstitution, presumably due to enhanced survival and homing of the HSCs transplanted. Preferential or exclusive long-term engraftment of the manipulated unit was detected in the vast majority or in half of the patients transplanted, respectively [27, 28];

finally, another pilot trial was based on *in vivo* treatment of recipients of single-CB unit grafts with sitagliptin, an inhibitor of the enzyme dipeptidyl peptidase-4 that has been shown to repress HSCs homing and engraftment through cleavage of the chemokine CXCL12 and of several critical hemopoietins [29, 30]. The results of this preliminary trial support the notion that systemic inhibition of dipeptidyl peptidase-4 may represent a simple, effective and relatively inexpensive method to enhance the engraftment of single CB units.

Numerous other molecules, including polyamine copper chelators (tetraethylene-pentamine [31]), antimicrobial cationic peptides (LL-37 [32]), histone deacetylase inhibitors (valproic acid [33, 34]), the small molecule, UM171, and its related pyrimidoindole derivatives [35], plant polyphenols (resveratrol [36]) and a synthetic OCT4 activator [37] have proven successful in improving HSC expansion and/or engraftment in preclinical models and are currently being validated, or will presumably be tested in the near future, in ad-hoc clinical trials.

In addition to extracellular ligands and synthetic or natural small molecules, an alternative strategy to effectively enhance the expansion and engraftment ability of hematopoietic stem cells is represented by transient modulation of cellular regulatory molecules that govern the quiescence/ survival/ self-renewal in these cells. For example, Lechman et al. [38] have recently demonstrated that knock-down of miR-126 through lentiviral sponge constructs results in strong expansion of murine and human long-term-repopulating HSCs. However, methods that result in permanent modification of the host cells genome pose significant safety problems-in particular the risk of so-called insertional mutagenesis, a consequence of vector integration, that may disrupt the normal expression or function of critical regulatory proteins and cause the development of malignancies [39]. A convenient alternative, when transient overexpression of a cellular regulatory factor is desired, is to exploit the properties of a segment of the HIV TAT protein rich in basic amino acid residues, termed protein transduction domain (PTD), that has been shown to facilitate the receptor-independent translocation of heterologous proteins across the surface membrane of target cells, including hematopoietic stem and progenitor cells [40, 41]. Using this approach, Krosl et al. [42] showed that incubation with a chimeric protein containing the TAT PTD fused to the HOXB4 homeoprotein induced a significant expansion of murine HSCs with long-term repopulating ability, comparable with that obtained through retroviral-mediated transduction of *HoxB4* [43]. Based on this evidence, and on the knowledge that the half-life of homeodomain-containing proteins is regulated at the post-translational level through ubiquitin-dependent degradation [44], in previous studies we generated a TAT-fusion protein containing a mutant version of HOXB4 [designated HOXB4(m)] lacking the LEXE motif in the homeodomain helix I that serves as a recognition site for the CUL4A ubiquitin ligase. Transduction of human mPB-CD34^+^ cells with this degradation-resistant TAT-HOXB4(m) strongly enhanced the accumulation of primitive progenitors and of stem cells capable of multi-lineage hematopoietic reconstitution of immunocompromised mice [45]. However, while the expression of HOXB4 in mPB-CD34^+^ is relatively low, its mRNA levels in CB-CD34^+^ cells are considerably higher in CB-CD34^+^ cells, and TAT-HOXB4(m) transduction failed to induce significant *ex vivo* expansion of these cells [45].

As an alternative protein transduction reagent to expand CB-CD34^+^ cells, we considered the polycomb group protein, BMI-1, a component of the Polycomb Repressor Complex (PRC) 1 and one major cell-intrinsic determinant of HSC proliferation and self-renewal [46–50]. Here we show that recombinant TAT-BMI-1 protein, produced in a prokaryotic system, efficiently penetrates in CB-CD34^+^ cells, accumulates in their nuclei and drives the expansion *in vitro* of primitive myeloid progenitors as well as of HSCs that display long-term repopulation potential in immunodeficient mice.

## RESULTS

### Experimental design

**Figure 1:**
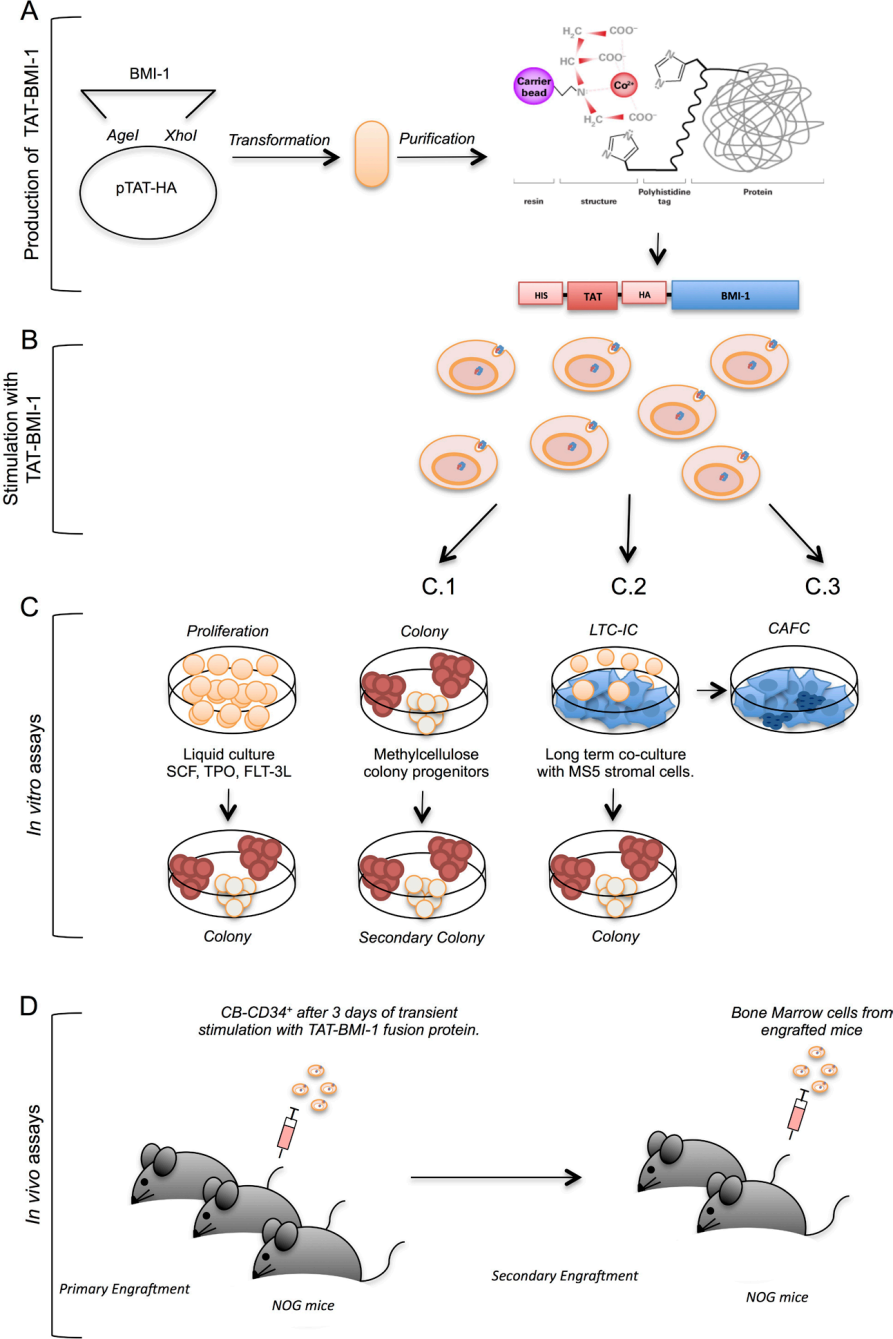
Experimental strategy This figure depicts the experimental workflow: (**A**) TAT-fusion proteins were produced in bacteria using the pTAT-HA plasmid, and purified by affinity chromatography; (**B**) the purified TAT-fusion protein were used to stimulate primary CB-CD34^+^ cells in the presence of early-acting hemopoietins (SCF, FL, TPO); (**C**) the cells thus stimulated were maintained in cytokine-driven cultures to monitor the total cell expansion and the numbers of colony-forming cells in these cultures (C.1), as well as the presence of self-renewing progenitors through secondary colony assays (C.2), or assayed in stromal co-cultures (C.3) to assess numbers of early myeloid progenitors (CAFCs); (**D**) CB-CD34^+^ cells treated with TAT-fusion proteins were assayed *in vivo* by xenotransplant of severely immunocompromised (NOG) mice following sublethal myeloablation with busulfan, and the engraftment of human cells was monitored in the blood and bone marrow of these animals by flow-cytometry; secondary transplants were performed to measure the presence of self-renewing long-term hematopoietic stem cells.

The experimental strategy is depicted in Figure 1. Briefly, the *BMI-1* coding sequence was inserted into the pTAT-HA vector downstream of, and in frame with, a sequence encoding a 6xHis tag, the HIV-TAT protein transduction domain and an HA tag (Figure 1A). These three motifs enable, respectively, efficient affinity purification of the chimeric protein through the 6xHis tag, entry into eukaryotic cells via the TAT PTD, and identification by Western blotting with high-affinity antibodies specific for the HA peptide. The fusion protein was produced in bacteria, purified as detailed below, and used to stimulate umbilical cord blood-derived CD34^+^ growing in the presence of cytokines that induce their proliferation (Figure 1B). After 3 days of incubation with TAT-BMI-1, or with the control protein TAT-GFP, the cells were subjected to *in vitro* (Figure 1C) and *in vivo* assays (Figure 1D) to assess whether exposure to TAT-BMI-1 enhanced the expansion of the most immature subset, including *bona fide* HSCs, as well as hematopoietic progenitors, compared to TAT-GFP.

### Purification of recombinant TAT-BMI-1

**Figure 2:**
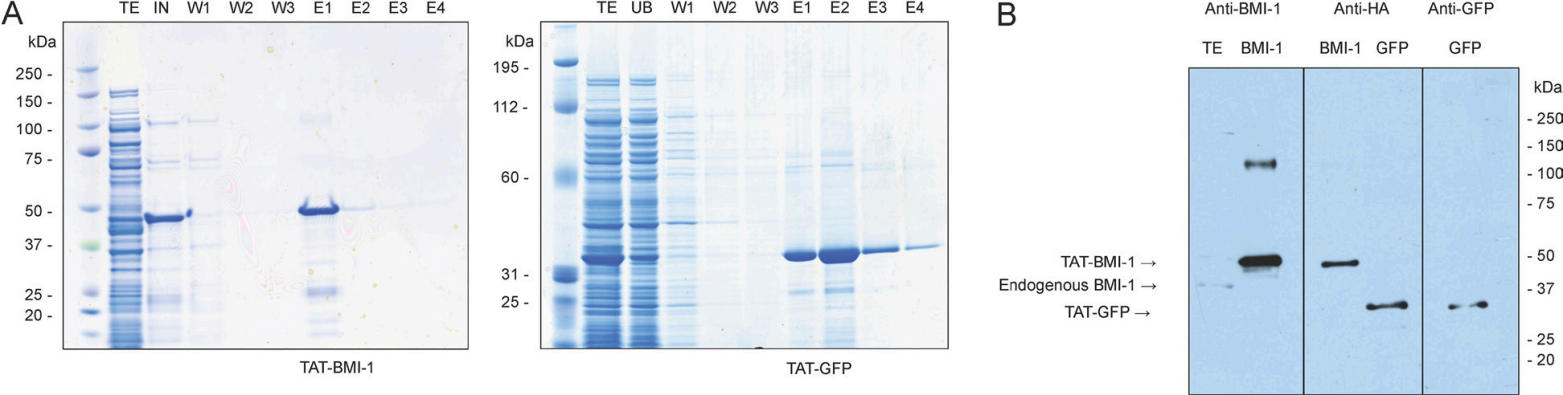
Affinity purification of TAT-BMI-1 and TAT-GFP recombinant proteins (**A**) Coomassie staining of NUPAGE 4–12% gels of total induced bacterial extract (TE), input (IN), unbound (UB) wash (W) and Imidazole elution (E). (**B**) Western Blotting of purified TAT-proteins, identified by specific antibodies against the HA epitope or for BMI-1 and GFP. A total extract from K562 shows the presence of native BMI-1 at 36.9 KDa compared to the TAT-BMI-1 at 45 KDa.

The *BMI-1* coding sequence was amplified by RT-PCR from RNA extracted from human CD34^+^ cells, sequenced and inserted into the pTAT-HA vector as detailed in Materials and methods. The presence of the 6xHis-TAT-HA moiety increased the molecular mass of the BMI-1 chimeric protein from 36.9 to 45 KDa, and raised its pI from pH 8.9 to 9.1. Large amounts of recombinant protein were produced upon induction in BL21 (OmpT and Lon protease negative) bacteria, and accumulated in the inclusion body fraction (Figure 2A). Therefore, affinity purification was performed under denaturing conditions with guanidium/HCl and urea. For this procedure the *HisPur Cobalt Resin* was used, as it is more resistant to leaching and requires lower amounts of imidazole to elute the protein. A TAT-GFP chimeric protein was also produced to be used as a control. This protein did not accumulate in inclusion bodies, but was found in the soluble fraction of bacterial extracts, thereby enabling affinity purification in native conditions (Figure 2A). Both TAT-proteins were highly purified as shown by Coomassie blue staining (Figure 2A), and could be detected using antibodies specific for the native proteins or for the HA tag (Figure 2B).

### Cell entry, nuclear localization, accumulation kinetics and activity of TAT-BMI-1

**Figure 3:**
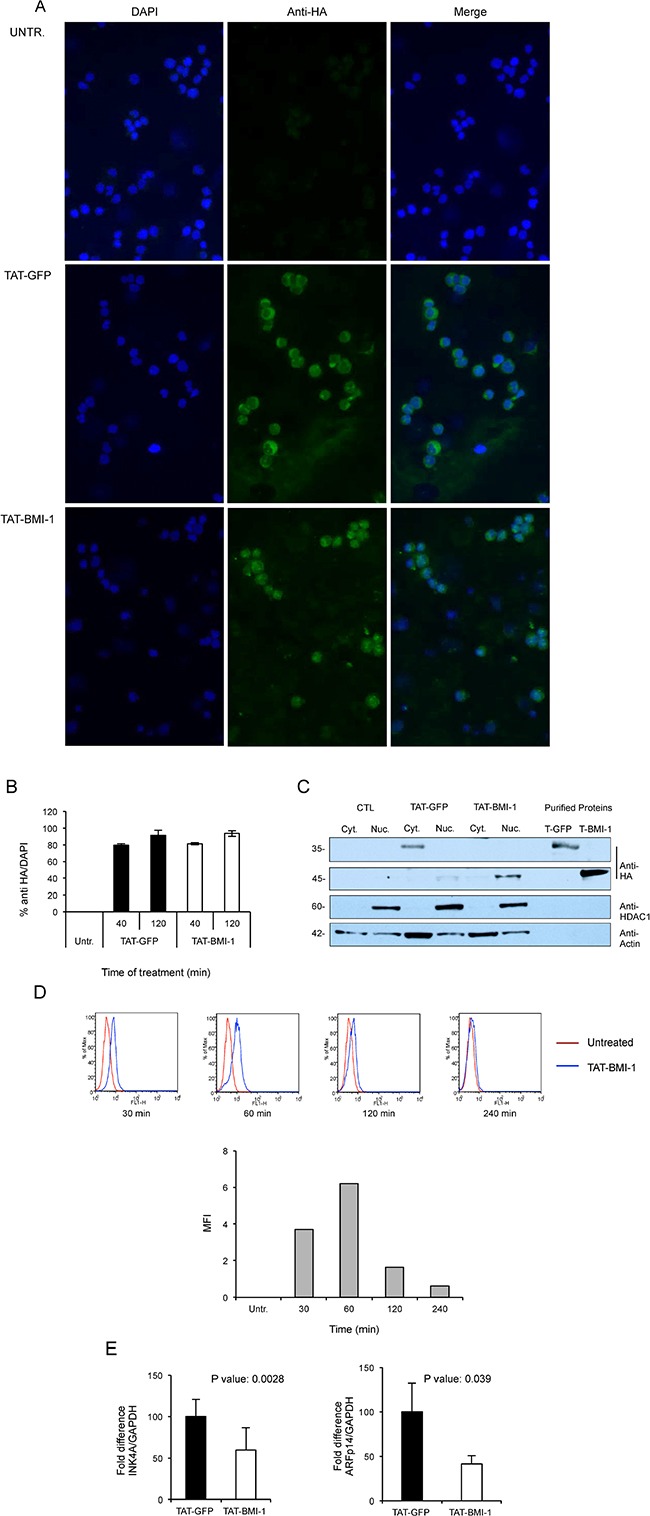
Uptake of TAT-proteins by cord blood derived CD34^+^ and K562 cells (**A**) Purified TAT-GFP or TAT-BMI1 proteins were added to cultures of CD34^+^ cells at a final 100 nM concentration for 40 min at 37°C. Cytospins were fixed and TAT-proteins were detected by indirect immunofluorescence with rabbit anti-HA and anti-rabbit Alexafluor 488 antibodies as detailed in Materials and Methods; cell nuclei were counter-stained with DAPI. (**B**) Uptake of TAT-GFP and TAT-BMI-1 in CB-CD34^+^ cells after 40 and 120 minutes of incubation. Treatment and immunofluorescence were performed as described above, and the percentage of HA-positive/DAPI-positive cells was determined using ImageJ. (**C**) Subcellular localization of TAT-proteins. K562 cells (2 × 10^5^/ml) were incubated with 100 nM TAT-fusion proteins for 20 min. Cytoplasmic and nuclear extracts were prepared as described in the materials and methods section, and analyzed by western blotting using an anti-HA antibody to reveal the TAT-fusion proteins, as well as anti-HDAC1 and anti-actin as markers of the nuclear and cytosolic protein fractions respectively. As it can be observed, TAT-GFP accumulates in the cytosolic fraction and TAT-BMI-1 in the nuclear fraction. As controls, 10 ng of affinity-purified TAT-GFP (T-GFP) and TAT-BMI-1 (T-BMI1) were also loaded. (**D**) Accumulation kinetics of TAT-BMI-1. K562 cells were incubated with 100nM protein and collected after 30, 60, 120 and 240 min. After intracellular staining with anti-HA antibody and Alexafluor 488-conjugated secondary antibody, FACS analysis was performed on a BD FaCScan (Beckton-Dickinson, Milan, Italy). In the top part of the panel, blue histograms represent TAT-BMI-1-treated cells compared to (red histograms) untreated cells. The mean fluorescence intensity (MFI) values determined at each time point, minus the MFI of untreated cells, are shown in the graph in the lower part of the panel. (**E**) TAT-BMI-1 down-regulates INK4A expression. 1 × 10^5^ CD34^+^ cells were stimulated for 3 days with repeated additions of 10 nM TAT-BMI-1. RNA was extracted and the INK4A and ARF mRNA levels were analyzed by RT-Q-PCR using GAPDH as a normalizing gene.

To test the ability of the TAT-BMI-1 to effectively enter target cells and migrate to the nucleus, CB-CD34^+^ cells were incubated for 20, 40 and 120 mins (Supplementary Figure 1) with either TAT-BMI-1 or TAT-GFP and then analyzed by immunofluorescence with an anti-HA antibody. As shown in Figure 3A (bottom panels), S1 and 3B, over 80% of the cells staining positive for TAT-BMI-1, that localized predominantly in the nuclei, whereas TAT-GFP accumulated mainly in the cytoplasm (Figure 3A).

To gain independent evidence of their subcellular localization, in parallel experiments TAT-BMI-1 or TAT-GFP were added to K562 cells and, after 20 mins of incubation, cells were harvested and cytoplasmic and nuclear extracts prepared and analyzed by Western blotting. TAT-BMI-1 was found in the nuclear fraction whereas TAT-GFP was instead in the cytoplasmic extract (Figure 3C), thus confirming the results shown in Figure 3A.

To evaluate the kinetics of intracellular accumulation of TAT-BMI-1 protein, K562 cells were cultured in the presence of 100nM fusion protein; after 30, 60, 120 and 240 min cells were collected, permeabilized and stained with anti-HA antiboby. FACS analysis shows a peak of accumulation after 60 min of culture with TAT-BMI-1, followed by a progressive decline of the intracellular levels of the protein (Figure 3D). Based on this observation, a schedule consisting of 4 additions/day of TAT-BMI-1 to the cultures was designed, in order to provide a continual supply of fresh protein to the cells throughout the treatment window.

To confirm the functional activity of TAT-BMI-1, the expression of the BMI-1 target gene, *CDKN2A*, encoding the CDK4/6 inhibitor p16^Ink4A^ and the p53 stabilizer, p14^ARF^, was measured by Q-RT-PCR in primary CB-CD34^+^ cells following 3-day treatment with TAT-BMI-1 or TAT-GFP. As shown in Figure 3E a significant, consistent, decrease in the levels of the transcripts for both *Ink4A* and *ARF* was observed in TAT-BMI-1-treated cells compared to those exposed to TAT-GFP, indicating that not only TAT-BMI-1 efficiently penetrates into CD34^+^ cells and achieves nuclear localization, but also retains its biological activity.

### TAT-BMI-1 induces expansion of umbilical cord blood-derived early hematopoietic progenitors *in vitro*

**Figure 4:**
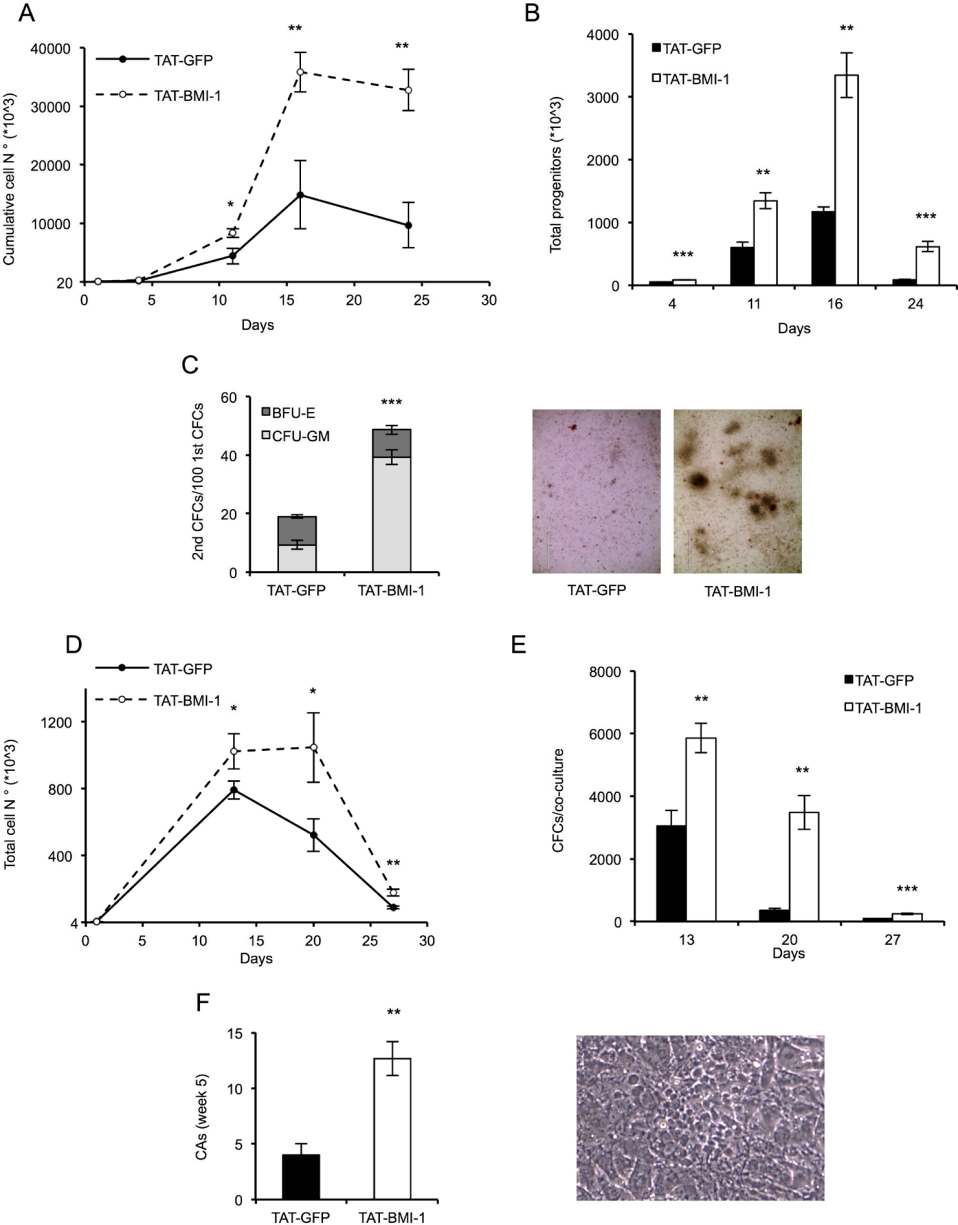
TAT-BMI-1 stimulation of CD34+ cell expansion *in vitro* (**A**) CD34^+^ cells (1–2 × 10^4^/ml) were plated in StemMACS HSC Expansion Medium with StemMACS HSC Expansion Cocktail. During the first 3 days of culture, TAT-BMI-1 or the control TAT-GFP protein were added 4 times a day at a 10 nM concentration. At the time intervals indicated, the cells were counted and re-plated in fresh medium at equal cell densities. Cumulative cell expansion is shown. All assays were performed in triplicate. (**B**) Colony-forming cell (CFC) assay was performed on cells from the cytokine-driven cultures at day 4, 11, 16 and 24. 100 (d. 4), 200 (d. 11), 300 (d. 16) or 600 (d. 24) cells/well were plated in triplicate assays in semisolid *StemMACS HSC-CFU* medium complete with cytokines. Colonies were scored after 2 weeks and total numbers of progenitors was normalized to the total cell number in the relevant culture at the time of plating. (**C**) Secondary colony assays were performed by collecting and re-plating in complete StemMACS HSC-CFU medium cells from primary colony assays (Figure 4B) set up with 100 cells/well from day 4 of liquid cultures. After 2 weeks colonies were scored for either BFU-E or CFU-GM morphology. A representative image of secondary colonies derived from TAT- GFP or TAT-BMI-1-treated cultures is shown. (**D**) Long term stromal co-cultures. 4 × 10^3^ cells/well (in triplicate), collected from cytokine-driven cultures after 3 days of treatment with the recombinant TAT-fusion proteins, were transferred onto monolayers of MS5 stromal cells and cultured in Myelocult medium with 1 μM hydrocortisone. At 13, 20 and 27 days the cultures were demi-depopulated, and the suspension-growing cells counted. The total numbers of cells/culture were calculated. (**E**) Colony-forming cell (CFC) assay were performed at day 13, 20 and 27 with cells collected from the MS5 stromal co-cultures. Colonies were scored as described above and total numbers of progenitors/culture were calculated based on the cell numbers of the relevant co-cultures. (**F**) Cobblestone area-forming cells (CAFCs) were scored in the stromal co-cultures after 5 weeks of culture. A cobblestone area (CA) is defined as a cluster of at least five small, non refractile cells that grow underneath or within the stromal layer. A representative image of cobblestone area derived from TAT-BMI-1 treated cells is shown. The average values of triplicate samples of representative experiments are shown in all panels, with the indication of SD values. *T*-test were performed to assess the statistical significance. *= *P* < 0.05; **= *P* < .005; ***=*P* < 0.0005

In these experiments, CB-CD34^+^ cells were plated in triplicate cultures in serum-free medium supplemented with a cocktail of hemopoietins including SCF, FLT3L and TPO, known to induce expansion of hematopoietic stem and progenitor cells [51]. Recombinant TAT-BMI-1 or control TAT-GFP (both at 10 nM final concentration) were added to the cultures 4 times a day for three days, in order to ensure the persistence of effective levels of the proteins. The cells were then maintained in culture in the presence of the cytokine cocktail and at regular intervals, counted and re-plated in fresh medium. Aliquots of the cultures at each time point were used for clonogenic assays in methylcellulose-containing medium, to assess the number of myeloid progenitors. As shown in Figure 4, a substantial increase both in total cell number (panel A) and in the number of colony-forming cells (panel B) was observed in TAT-BMI-1-treated cultures compared to those incubated with TAT-GFP. To test whether TAT-BMI-1 stimulated self-renewal of primitive progenitors, secondary CFC assays were also performed, that showed a considerable increase in the number of secondary colonies (in particular of CFU-GM) yielded by cells exposed to TAT-BMI-1 compared to those derived from control cultures (Figure 4C).

To assess the effect of TAT-BMI-1 on highly immature hematopoietic progenitors, CB-CD34^+^ cells were assayed in stromal co-cultures with MS-5 cells. These co-cultures mimic some of the features of the stem cell-niche interaction, and sustain prolonged *in vitro* generation of hematopoietic cells from primitive progenitors [52]. In these assays CB-CD34^+^ cells exposed to TAT-BMI-1 gave rise to significantly higher numbers of total cells (Figure 4D) and clonogenic progenitors (Figure 4E) than those treated with TAT-GFP. Of particular relevance, a 3-fold increase in the number of week 5 cobblestone area-forming cells (CAFCs), which represent the most primitive hematopoietic progenitors that can be assayed *in vitro*, was observed in the co-cultures established with TAT-BMI-1-treated CB-CD34^+^ cells than in the control TAT-GFP-treated counterpart (Figure 4F).

### TAT-BMI-1 induces expansion of CB-HSCs capable of long-term repopulation

To determine whether the increase in hematopoietic progenitors induced by TAT-BMI-1 was mirrored by a comparable increase in the frequency of hematopoietic stem cells, we performed xenotransplants in severely immunocompromised NOD-*scid* IL2Rγ*^null^* (NOG) mice, presently considered the most sensitive system to assess human hematopoietic stem cell activity [53, 54].

For engraftment of human CD34^+^ cells, partial myeloablation of the recipient mice is required. This is typically achieved by sublethal irradiation (< 400 Gy). However, busulfan treatment has emerged as a convenient and more flexible alternative to irradiation for conditioning of NOG mice, as it has been shown to induce lower mortality and milder weight loss and reduction in blood counts, while resulting in similar or superior engraftment of transplanted HSCs [55–57]. In preliminary experiments (not shown), we determined that using a conditioning regimen based on dual intraperitoneal busulfan administration (25 mg/Kg), a robust chimerism (> 30%) could be detected in the peripheral blood of recipient mice up to 30 weeks following the transplant of human CB-CD34^+^ cells. Therefore, the NOG mice used in these assays were conditioned with a double dose of 25 mg/Kg of busulfan. After 3–4 days of observation, the mice were injected with CB-CD34^+^ cells (3 × 10^4^ cells/mouse) that had been pre-treated either with TAT-BMI-1 or TAT-GFP for 3 days as detailed above.

**Figure 5:**
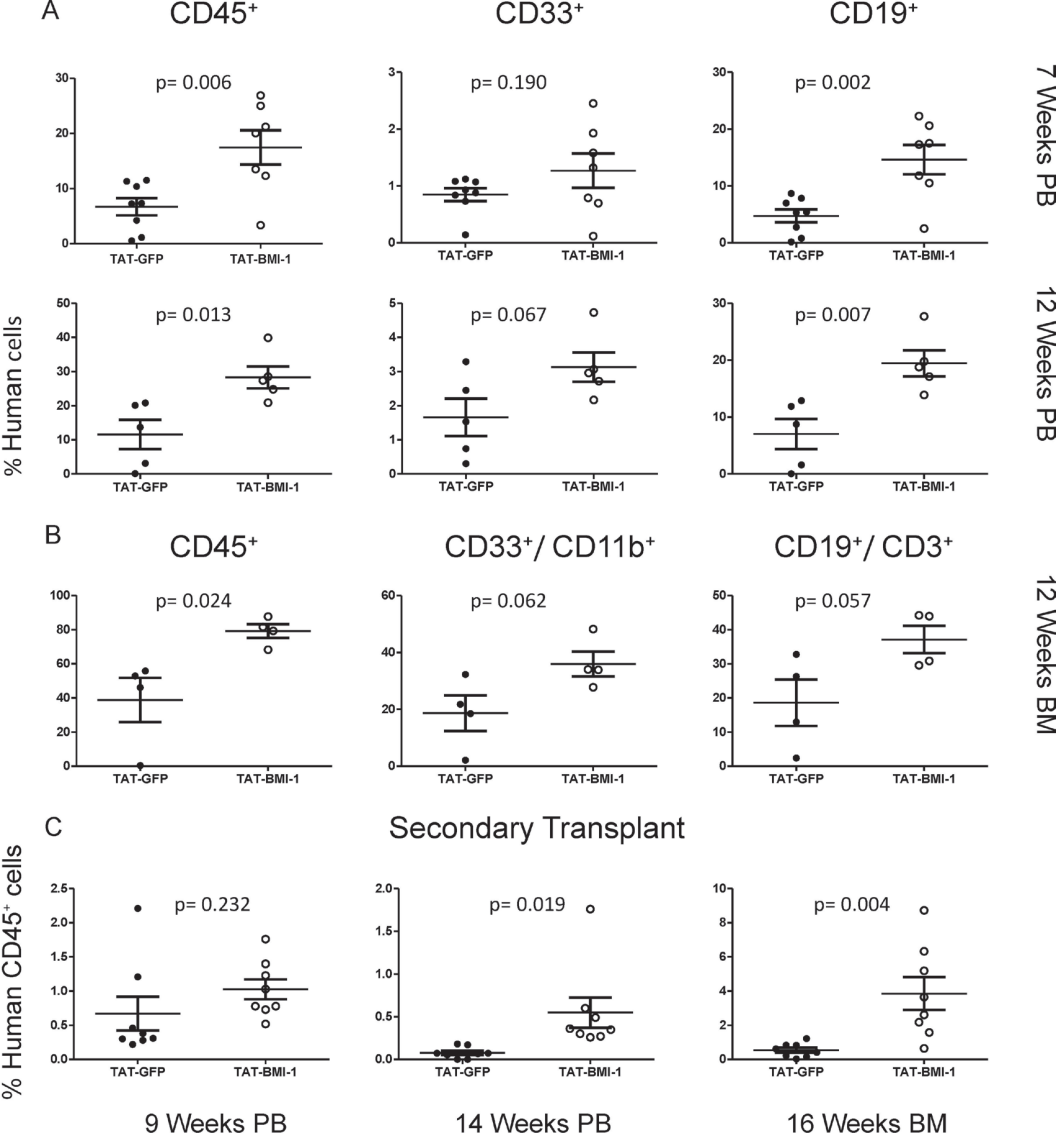
TAT-BMI-1 pretreatment of CB-CD34^+^ cells enhances hematopoietic engraftment in NOG mice (**A**) The percentage of total human leukocytes (CD45^+^), myeloid (CD33^+^) and B-lymphoid (CD19^+^) cells in peripheral blood of NOG mice transplanted with TAT-GFP or TAT-BMI-1-treated CD34^+^ cells was analysed by flow cytometry 7 and 12 weeks after the injection. (**B**) The percentage of total human leukocytes (CD45^+^), myeloid (CD11B^+^,CD33^+^) and B-lymphoid (CD19^+^, CD3^+^) cells in the bone marrow of NOG mice transplanted with TAT-GFP or TAT-BMI-1-treated CD34^+^ cells was analysed by flow cytometry 12 weeks after injection. (**C**) The percentage of total human CD45^+^ cells was analysed by flow cytometry in peripheral blood (weeks 9 and 14 post-transplant) and bone marrow (week 16 post-transplant) of NOG mice transplanted with bone marrow from the primary recipients shown in panel B.

Throughout the experiments the mice were monitored regularly, and weighed twice per week. Blood samples were collected from the facial vein at 7 and 12 weeks post-transplant, to assess the presence of human hematopoietic cells by flow cytometry. These analyses revealed a > 2-fold increase in the percentage of circulating human leukocytes, that included predominantly lymphocytes but also a significant proportion of myeloid cells, in the peripheral blood of mice transplanted with TAT-BMI-1-treated CB-CD34^+^ cells compared to the recipients of control cells (Figure 5A, Supplementary Figure 2). Between week 7 and week 12, 3 mice of the TAT-GFP cohort and 2 of the TAT-BMI-1 cohort were found dead without any obvious prior signs of disease.

Four mice of each cohort were sacrificed on week 12. Bone marrow analysis highlighted a higher percentage of human cells than in peripheral blood, and the comparable abundance of myeloid and lymphoid cells. Consistent with the data obtained from peripheral blood, a 2-fold higher engraftment rate was observed in the bone marrow of mice injected with TAT-BMI-1-treated CB-CD34^+^ cells compared to controls (Figure 5B, Supplementary Figure 3). One mouse of each group was preserved for further monitoring. The TAT-GFP mouse was found dead on week 18, whereas the TAT-BMI-1 animal was monitored until week 20 and then sacrificed. No signs of leukemia nor of other diseases were observed at the autopsy.

The bone marrow of the sacrificed mice was used for secondary transplants, and the presence of human leukocytes in the peripheral blood of secondary recipients was monitored at week 9 and week 14 post-transplant. As expected, secondary engraftment rates were lower in both populations (Figure 5C, left panel, Supplementary Figure 4), but a much stronger decline in the fraction of human cells occurred in control mice than in the TAT-BMI-1 cohort (Figure 5C, middle panel). This was confirmed by bone marrow analyses performed 16 weeks after transplant (Figure 5C, right panel) where an average 0.5% human CD45^+^ cells were detected in TAT-GFP mice compared to 3.8% in the TAT-BMI-1 counterpart, indicating that the TAT-BMI-1-treated CB-CD34^+^ were enriched in long-term repopulating stem cells. As in the primary recipients, no signs of leukemia were detected in the mice subjected to secondary transplants throughout the experiments.

## DISCUSSION

In this study we have shown that a relatively brief *in vitro* treatment with the recombinant chimeric protein, TAT-BMI-1, can induce expansion of human cord blood-derived long-term repopulating stem cells and early myeloid progenitors.

BMI-1, a polycomb protein family member, is acknowledged as a critical regulator of self-renewal in both normal and malignant stem cells of different origins, with particular regard to the hematopoietic system. In the symmetrical division of a self-renewing stem cell, it is essential that both daughter cells conserve the same epigenetic landscape as the parental cells, in order to inherit the full repertoire of molecular features that characterize stem cells. A variety of epigenetic regulators, among which the polycomb group proteins, are considered crucial players in maintaining such ‘epigenetic memory’, and hence stem cell identity, through stem cell divisions.

BMI-1 is a component of the polycomb repressor complex (PRC)1. In *Drosophila*, PRC1 is composed of five core subunits, designated Polycomb (Pc), Polyhomeotic (Ph), Posterior sex combs (Psc), Sex Comb on Midleg (Scm) and Sex combs extra (Sce/Ring). In human cells, each of these proteins correspond to classes of multiple paralogs – termed PCGF, PHC, CBX, SCML and RING respectively-that can assemble in various combinations to form functionally distinct PRC1 complexes [58]. BMI-1 (PCGF4) is the best characterized PRC1 member, and has been implicated by numerous studies as a crucial determinant of HSC self-renewal. Ablation of the *Bmi-1* gene in mice resulted in a severe post-natal reduction of HSCs, accompanied by complete loss of their self-renewal ability [47] whereas enforced expression strongly enhanced HSC self-renewal and promoted their *ex vivo* expansion [49]. Consistently, *BMI-1* silencing in human CB-CD34^+^ cells abolished their growth in culture and induced apoptosis [50] whilst its retroviral-mediated enforced expression allowed the long-term *ex vivo* maintenance of early hematopoietic progenitors and of HSCs with extensive self-renewal potential [48]. The fact that overexpression of *BMI-1* augments HSC self-renewal suggests that the levels of this factor in steady-state hematopoiesis may be limiting for PRC1 activity, or for the assembly of specific PRC1 complexes able to enhance self-renewal, and therefore that a transient increase in its amounts would not be redundant but may effectively aid *ex vivo* expansion of HSC.

As the safest methodology to achieve such a transient overexpression we chose protein transduction. This is an effective method to introduce exogenous polypeptides in cultured cells, and has been exploited to deliver a number of regulatory proteins to CD34^+^ cells to induce their expansion *in vitro* [45, 59–61]. Protein transduction avoids potential risks associated with introduction of foreign genetic material into the target cells and, more importantly, is intrinsically limited in the duration of its effects by the finite intracellular half-life of the protein transduced. We first explored the feasibility of this approach and established that, despite its accumulation in inclusion bodies, adequate amounts of TAT-BMI-1 could be purified in a relatively easy manner using affinity chromatography (Figure 2). We then verified that the fusion protein efficiently transduced CD34^+^ cells (Figure 3A, 3B), localized to the nucleus (Figure 3A, 3C) and down-regulated the expression of *INK4A* and *ARF*, major BMI-1 targets whose products are regarded as prominent inhibitors of HSC self-renewal (Figure 3E). Finally, we showed that treatment of CB-CD34^+^ cells with TAT-BMI-1 resulted in an over 3-fold higher expansion of the total cell population compared to control cells, mirrored by a comparable increase in the frequency of myeloid progenitors, in cytokine-driven cultures (Figure 4A–4B). Consistently, a significant increase in total cell numbers, as well as in those of committed (CFU-C) and more primitive (CAFC) progenitors [62] was observed in stromal co-cultures (Figure 4D–4F). More importantly, xenotransplants in sublethally myeloablated NOG mice showed that short exposure of CB-CD34^+^ cells to TAT-BMI-1 was sufficient to enhance by approx. 2-fold their rate of lympho-myeloid engraftment in primary recipients, (Figure 5A–5B), and by almost 7-fold the secondary engraftment (Figure 5C), indicating a robust increase in the frequency of HSCs capable of long-term hematopoietic reconstitution, consistent with the findings of Rizo *et al*. [48] in BMI-1-transduced CB-CD34^+^ cells.

Using an approach similar to that described in this paper, Kajiume et al. [63] investigated the effects of protein transduction of mouse Bmi-1 and of its paralog, Mel-18/ PCGF2, fused to a PTD, on adult bone marrow HSCs of murine origin. These authors observed an over 10-fold increase in the total cell expansion *in vitro*, and a more moderate enhancement (≤ 50%) in the engraftment of TAT-Bmi-1-treated cells in syngeneic mice. In our conditions, the difference in cell expansion triggered by human TAT-BMI-1- vs. TAT-GFP was lower, however it must be considered that cord blood-derived CD34^+^ cells are of fetal origin and display a considerably stronger intrinsic proliferative potential - as shown by the over 1,800-fold increase in the number of total cells over two weeks of culture, compared to a 500-fold expansion of mouse BM cells [63]. Conversely, the difference in engraftment between TAT-BMI-1 and TAT-GFP-treated CB-CD34^+^ cells was significantly higher than that measured by Kajiume et al.

Kajiume et al. also reported the death of 80% of the recipients of TAT-Bmi-1-treated cells within 15 weeks from the transplant, and postulated that this may reflect the occurrence of leukemia. Although unlikely, owing to the short duration of TAT-BMI-1 treatment, such a possibility cannot be excluded in principle, as BMI-1 has been implicated in tumorigenesis and is known to support the self-renewal of leukemic stem cells [46, 64]. However, in our assays the deaths in the TAT-BMI-1 cohort were less numerous than in the TAT-GFP cohort, and no signs of leukemia were detected in any of the mice subjected to primary xenotransplants, nor in the secondary recipients that were monitored for 16 weeks prior to their sacrifice for bone marrow analysis. This strongly suggests that no leukemic cells were generated by exposure to TAT-BMI-1, since the presence of even a minute fraction of transformed cells in the TAT-BMI-1-treated population would have given rise to a full-blown leukemia, if not in primary at least in the secondary recipients through the long follow-up period.

In the light of a recent report [65] showing that deletion of the 90-AA-long proline- and serine-rich (PS) carboxyl-terminal domain of BMI-1 significantly extended its half-life in mammary epithelial cells and human fibroblasts without affecting its growth-promoting and senescence-inhibitory effects, we tested whether deletion of this C-terminal domain in TAT-BMI-1 (TAT-BMI-1 235, Supplementary Figure 5A) may similarly enhance its biological activity in CB-CD34^+^ cells. However, despite its efficient entry into the cells and accumulation in the nucleus (Supplementary Figure 5B), this truncated fusion protein yielded a lower total cell expansion than full length TAT-BMI-1, and failed to induce a significant CFC expansion (Supplementary Figure 5C–5D), possibly reflecting either cell-type-specific or species-specific differences in the functional requirement for the PS domain. This approach was therefore not pursued further.

Despite considerable advances in the development of *ex vivo* expansion strategies and the promising results of ongoing clinical trials, the discovery of novel factors able to support safe and effective expansion of cord blood-derived hematopoietic stem and progenitor cells remains of considerable relevance. Even in the most successful trials [24, 26], despite the large excess of CD34^+^ cells in the expanded unit, in over one-third of the cases long-term engraftment derived entirely from the non-manipulated unit, and it cannot be excluded that loss of stem cell activity may have occurred in the expanded specimens. The most prominent property of BMI-1 is that of promoting self-renewal and maintaining the multipotency of a variety of somatic stem cells including HSCs. The effectiveness of this factor in enhancing the expansion of CB-derived HSCs and early progenitors in culture has been confirmed in this study. It is thus conceivable that transduction with BMI-1 protein, alone or in combination with other treatments used to achieve *ex vivo* CB-HSC expansion, may help circumventing such potential loss of “stemness” and ensure the maintenance of engraftment potential of CB-CD34^+^ cells through the expansion procedures. Recombinant TAT-BMI-1 may therefore represent a novel, valuable reagent for *ex vivo* expansion of CB-HSC for therapeutic purposes.

## MATERIALS AND METHODS

### Cells and mice

Human CB-CD34^+^ primary cells (Poetics, Lonza) were cultured in StemMACS HSC Expansion Medium (Miltenyi Biotech) supplemented with StemMACS HSC Expansion Cocktail (Miltenyi Biotech), containing stem cell factor (SCF), FLT3 ligand (FLT3L) and thrombopoietin (TPO). K562 cells were cultured in RPMI supplemented with 10% FBS, glutamax and pen/strep (all from Life Technologies). MS5 stromal cells were cultured in alpha MEM (Lonza) supplemented with 10% FBS, glutamax and pen/strep. All cells were maintained at 37°C in 5% CO_2_.

Female mice 6–8 weeks old, NOG strains were purchased from Harlan and maintained in specific pathogen-free (SPF) conditions prior to and during the *in vivo* experiments. The xenotransplant procedures in immunodeficient mice were approved by the Institutional Animal Welfare Committee of Biogem, and conformed in all their aspects to the institutional guidelines that comply with national and international laws and policies. The engraftment experiments were conducted according to the published guidelines for the welfare and use of animals [64]. All animals were monitored for a decrease in physical activity or other signs of disease or distress; severely ill animals were euthanized by CO_2_ asphyxiation.

### Construction of the expression vectors pTAT-GFP and pTAT-BMI-1

The construct pTAT-HA [66] contains a 6xhis-TAT-HA cassette at the N-terminal end, which can be exploited for purification via immobilized metal ion affinity chromatography (IMAC). In the sequence of the recombinant fusion protein, this motif is followed by a sequence of 11 amino acids from the HIV TAT protein, termed protein transduction domain (PTD), which has been shown to facilitate entry into the target cells [66]. Downstream of the TAT PTD, a short amino acid motif from the influenza virus hemagglutinin (HA) provides an additional tag for recognition of the recombinant fusion protein with specific anti-HA antibodies.

To construct the TAT-GFP expression vector, EGFP cDNA was amplified from pIRES2-EGFP (Clontech) by PCR using the following oligonucleotides:

FWD: CTCGAGATGGTGAGCAAGGGCGAGGA;

REV: CTTCGAATTCTTACTTGTACAGCTCGTCCAT GCCG.

The cDNA was subsequently cloned into in the pTAT-HA vector using the XhoI and EcoRI restriction sites. For the construction of the TAT-BMI-1 vector, BMI-1 cDNA was amplified from mRNA from human CD34^+^ cells with the following primers:

FWD: ACCGGTATGCATCGAACAACGAG

REV: CTCGAGTCAACCAGAAGAAGTTGCTG and was then introduced into the AgeI and XhoI sites of pTAT-HA.

The truncated form of BMI-1 (BMI-1 235) was constructed by amplifying the sequence encoding its amino acids 1–235 from mRNA from human CD34^+^ cells using the following primers:

FWD: ACCGGTATGCATCGAACAACGAG

REV: CTCGAGTCACTTCATTCTTTTACAAGTAGGTC and introduced into the AgeI and XhoI sites of pTAT-HA.

The sequence of all constructs was verified prior to their use (Eurofins, MWG Biotech).

### Expression and purification of TAT-fusion proteins

The pTAT-GFP and pTAT-BMI-1 plasmids were transformed into *E. coli* BL21 (DE3, OmpT and Lon protease deficient), and induced with 0,25 mM Isopropril-β-thio-galattoside (IPTG, Sigma) for 2 h in agitation at 37°C. The bacterial cultures were centrifuged (3,000 × g for 15 min at 4°C) and the pellets were resuspended in Lysis buffer, consisting of 50 mM Tris/HCl pH 8.0, 50 mM NaCl, 1 mM EDTA, supplemented with cellular protease inhibitors (*P8849;* Sigma) and subjected to 5 10-second cycles of sonication (Ultrasonic Cell Disruptor *XL*; MICROSON, setting #8) at 4 °C. The TAT-BMI-1 protein was exclusively evident in the insoluble inclusion bodies, which were washed with lysis buffer and solubilized in Guanidium buffer (6M guanidine/ HCl, 20 mM sodium phosphate pH 7.8, 500 mM NaCl, 10% glycerol). After removal of the insoluble fraction by centrifugation at 10,000g for 10 mins at 4°C, the supernatant was collected and subjected to metal affinity chromatography to purify the 6xHis tag-bearing fusion protein, with *HisPur Cobalt Resin* (Thermo Scientific) pre-equilibrated with 8 M Urea, 300 mM NaCl, 50 mM sodium phosphate pH 7.4. After application of the guanidine/HCl solubilized protein, the column was washed with 10 column volumes of equilibration buffer containing 10 mM imidazole, and then eluted with the same buffer containing 150 mM imidazole. The protein concentrations of the eluted fractions were determined by the Bradford colorimetric assay (*BIO-RAD protein assay*, Bio-Rad) by measuring its absorbance at 595nm with BSA as a standard protein. The fractions were analysed by 4–12% gel NuPAGE^®^ (Life technologies) and visualised using Coomassie Brilliant Blue G-Colloidal staining (Sigma) and by Western blotting as described below. The fractions enriched in TAT-BMI-1 were pooled and subjected to two rounds of dialysis against 500 volumes of phosphate buffered saline (PBS) at 4°C (for 16 and 6 hours respectively) and sterilized by UV light exposure, samples were exposed in petri dishes on ice at 50 cm from the UV source (240 nm) for 20 min. Aliquots were quantified and tested for sterility by incubation in culture medium for 48h at 37°C. To avoid protein aggregation, proteins were stored in aliquots in 10% glycerol at -20°C.

For TAT-EGFP protein, after sonication the soluble fraction was loaded onto cobalt affinity columns and eluted with 150 mM imidazole in Lysis buffer. After dialysis against PBS as described above, the recovered protein was sterilized by 0.2 μm filtration (cellulose acetate).

Western blotting analyses performed using primary mouse monoclonal anti-BMI-1 Clone 1H6B10G7 (Life Technologies), primary rabbit monoclonal anti-HA (ab9110, Abcam), primary rabbit monoclonal anti-GFP (G1544 Sigma) and goat anti-mouse/anti-rabbit HRP (Santa Cruz). Chemiluminescence was detected using the ECL reagent (Santa Cruz) and autoradiographic x-ray film.

Endotoxin levels in the purified TAT-fusion protein samples were measured using Limulus Amebocyte Lysate (LAL) assay kits (LAL PYROGENT™ and PYROGENT™Plus LONZA). The endotoxin concentrations detected in the TAT-GFP and TAT-BMI-1 preparations used in the experiments illustrated in this paper ranged between 0.12 and 0.5 Endotoxin Units (EU)/ml.

### Analysis of TAT-fusion proteins uptake and intracellular localization

Cryopreserved cord blood-derived CD34^+^ cells were thawed and plated at at 2 × 10^5^ cells/ml in StemMACS HSC Expansion Medium, supplemented with StemMACS HSC Expansion Cocktail. After over-night recovery from thawing, TAT-GFP or TAT-BMI-1 were added to the cells at a final concentration of 100 nM. Cells were collected after 20, 40 and 120 min, washed in cold PBS placed on a cytospin slide and centrifuged at 400 rpm for 4 min the *Shandon Cytospin* *3* centrifuge (Thermo Electron Corporation). The samples were fixed in ice-cold 4% paraformaldehyde for 20 min at 4°C in the dark, permeabilized with 0.5% Triton X-100 in PBS for 5 min and blocked with 1% BSA, 0.1% Triton X-100 in PBS, for 20 min. The slides were incubated with anti-HA (Abcam) primary rabbit antibody 1:500 overnight, washed and subsequently with anti-rabbit Alexafluor 488 1:200 (Life Technologies) for 1h in blocking buffer. The nuclei were stained with 10 ng/ml DAPI in PBS and images captured with a DFC 3000 G camera mounted on a Leica Microscope (DM IL LED) at a 20x magnification. Numbers of cells positive for BMI1 uptake were counted and divided by the number of nuclei present using Image J analysis.

2 × 10^6^ K562 cells were incubated in 5 ml of RPMI 10% FBS containing 100 nM TAT-GFP or TAT-BMI-1 for 20 mins at 37°C. Cytoplasmic and nuclear extracts were prepared as described by Chiarella et al. [67] using hypotonic lysis (containing 10 mM Hepes pH 7.9, 10 mM KCl, 0.1 mM EDTA, protease inhibitors (P8849 Sigma) for cytoplasmic proteins, and high-salt buffer (20 mM Hepes pH 7.9, 400 mM NaCl, 1 mM EDTA, protease inhibitors) to recover nuclear proteins. The enrichment of the cytosolic proteins was confirmed by Western blotting with mouse anti-actin (A4700 Sigma)1:10000; that of nuclear proteins by rabbit anti-HDAC1 1:10000 (H3284 Sigma). Recombinant 6xhis-TAT-HA-GFP and 6xhis-TAT-HA-BMI-1 were revealed with rabbit anti HA 1:10000. Detection was performed with goat anti-mouse or anti-rabbit HRP (DBA, Santa Cruz) (1:2000) followed by chemiluminescence using ECL reagent (DBA, Santa Cruz) and autoradiography.

### Kinetics of intracellular accumulation of TAT-BMI-1 protein

K562 cells were incubated with 100 nM TAT-BMI-1 protein as detailed above. After 30, 60, 120 and 240 min aliquots of the cells were collected, washed with cold PBS and fixed with cytofix (BD Biosciences) for 20 min. After washing, the cells were permeabilized with permawash 1× (BD Biosciences) for 10 min, resuspended in blocking solution (containing: permawash 1× (BD Biosciences) 10% FBS (Life Technologies) and 10% FcR Blocking Reagent, human (Miltenyi Biotech) for 45 min and incubated with anti-HA (Abcam) primary antibody (1:2000) for 45 min and with anti-rabbit (Alexa Fluor 488) secondary antibody. Flow-cytometric analysis was performed using a FACScan flow cytometer (Beckton Dickinson) and FlowJo software.

### RNA extraction, cDNA synthesis and Q-RT-PCR

Total RNA was prepared by TRIZOL (Life Technologies) lysis, followed by treatment with DNase I (RNase free, Promega), as described by Spina et al [68]. The purity and amounts of RNA were determined by measurement of the OD260/280 ratio. cDNA was synthesized from 1μg RNA using SuperScript III reverse transcriptase at 42°C and 2.5 μM random hexamers (Life Technologies). Q-PCR reactions were carried out in triplicate with the iQ SYBR green Supermix (Bio-Rad) according to the manufacturer's instructions and amplified using iQ5 Real time multicolor detection system (Bio-Rad). One cycle of 3 min at 95°C was followed by 45 cycles of 10 seconds at 95°C, 10 seconds at 60°C and 20 seconds at 72°C, and then melting curves were determined. The specificity of amplification was confirmed by the dissociation curves of the amplicons and size of the amplified product analyzed on 2% agarose gels. The PCR primers for human INK4A and for human p14ARF were designed based on the sequence of those described by Bracken et al [69]. The primers for GAPDH were those previously described [70]

INK4A: Fwd GAAGGTCCCTCAGACATCCCC; Rev CCCTGTAGGACCTTCGGTGAC

ARF: Fwd CCCTCGTGCTGATGCTACTG; Rev ACCTG GTCTTCTAGGAAGCGG;

GAPDH: Fwd CACCATCTCCCAGGAGCCAC; Rev TC ACGCCACAGTTTCCCGGA

Relative gene expression was determined using the comparative threshold cycles Ct method, normalizing for endogenous GAPDH primers [70]. The expression ratio was calculated as 2^−ΔΔCT^.

### CD34^+^ cells expansion assay

Cryopreserved cord blood-derived CD34^+^ cells were thawed and plated in triplicate at 1–2 × 10^4^ cells/ml in StemMACS HSC Expansion Medium supplemented with StemMACS HSC Expansion Cocktail. After over-night recovery from thawing, cells were treated with repeated additions (4/day) of 10 nM TAT-GFP or TAT-BMI-1 for 3 days. Subsequently cultures were maintained without further addition of TAT-protein, for several weeks and periodically (every 3–6 days) cells were counted and replated at equal cell concentrations. For counts, cells were stained with trypan blue (Life Technologies) and counted in a Bϋrker chamber.

### Clonogenic assays

For colony-forming cell (CFC) assays, aliquots of cells, deriving from the cytokine-driven cultures described above or from the stromal co-cultures detailed below, were plated in triplicate in 0.5 ml *StemMACS HSC-CFU complete with Epo Medium* (Miltenyi Biotech), composed of 1% methylcellulose in Iscove's MDM 30% FBS, 1% BSA, 2 mM glutamine, 0.1 mM 2-mercaptoethanol, 50 ng/ml SCF, 20 ng/ml GM-CSF, 20 ng/ml G-CSF, 20 ng/ml IL3, 20 ng/ml IL6, 3 U/ml Epo. 10^2^ cells/well were plated on day 4 of culture, increasing amounts (2, 3 and 6 × 10^2^) in the assays performed at later time points. Colonies were scored after 2 weeks with an inverted microscope (Leica DM IL LED) at 5× magnification.

### Stromal co-cultures

For long term stromal co-cultures, after 3 days of treatment with the recombinant proteins in liquid culture, 4 × 10^3^ cells/well were plated in triplicate wells onto a monolayer of MS5 stromal cells (kindly provided by Dr. J.J. Shuringa, University Medical Center Groningen, Groningen, The Netherlands), in Myelocult medium (StemCell Technologies) containing 1 μM hydrocortisone (Sigma). At weekly intervals half of the supernatant was removed for cell counting and replaced with fresh medium. The cells in the supernatant were cultured and used for CFC assays as described above. After 4–5 weeks of co-culture, cobblestone area forming cells CAFCs were scored with an inverted microscope (Leica DM IL LED ) at 20× magnification. A cobblestone area (CA) is defined as cluster of at least five round-shaped, non refractile (phase-dark) cells embedded in the stromal layer [71].

### Conditioning and transplantation of NOG mice

Female 6–8 weeks old, NOG mice were used for the CD34^+^ transplants. Conditioning was performed with two intra-peritoneal administrations (4 and 3 days prior to the transplant) of 25 mg/kg of body weight of busulfan (Sigma Aldrich, B2635), solubilized in 10% DMSO (Acros, 327182500), 90% Corn Oil (Sigma Aldrich C8267). 3 days after the second injection, cohorts of 8 mice were inoculated in the tail vein. Each mouse received 3 × 10^4^ CB-CD34^+^ cells pre-treated for 3 days with TAT-GFP or TAT-BMI-1 as described for the expansion assays, resuspended in 100 μl PBS. Control mice received 100 μl PBS.

For secondary transplants, the bone marrow samples from the four sacrificed mice of each cohort were pooled and 2.5 × 10^6^ cells/mouse were injected in two cohorts of 8-weeks old female NOG mice pre-conditioned with busulfan as described above.

### Flow-cytometric analyses of mouse blood and bone marrow

Prior to the analyses, erythrocytes were lysed with Red Blood Lysis Buffer (154 mM NH_4_Cl, 14 mM NaHCO_3_ and 0.1 mM EDTA). For flow cytometry analyses the following reagents were used: FcRBlocking Solution (BD Biosciences 553142); anti Human CD45 PerCp (BD Bioscience BMS45-9459-42); anti Human CD3 PE (BD Biosciences 555340); anti Human CD19 FITC (BD Biosciences 555412); anti Human CD33 PE (BD Biosciences 555450); anti Human CD11b FITC (BD Biosciences 562793) and, as an isotipic control, Simultest (BD Biosciences 342409). Flow-cytometric analysis was performed using the FACScan Beckton Dickinson and FlowJoX software. Blood cell counts were performed with the VetScanHM5 (ABAXIS). After 12 weeks, four mice of each cohort were sacrificed by cervical dislocation. Bone marrow from femur and tibia of these animals was sampled as described by Soleimani and Nadri [72] and collected in DMEM 10% FBS, 10 U/ml Heparin for flow-cytometry analysis and secondary transplant.

To monitor the secondary engrafment, flow-cytometric analyses of human CD45^+^ cells in the peripheral blood of the secondary recipients were performed 9 and 14 weeks after the transplant. On week 16, the mice were sacrificed and their bone marrow was collected and analysed.

### Statistical analysis

*P*-values were obtained by applying a two-tailed, unpaired *t*-test using Microsoft Excel and GraphPad Prism 5. *P*-values < 0,05 were considered significant.

## SUPPLEMENTARY MATERIALS FIGURES AND TABLES


