# Supplementary material for: Recombinant TAT-BMI-1 fusion protein induces ex vivo expansion of human umbilical cord blood-derived hematopoietic stem cells
Source: Oncotarget. 2017 Feb 7;8(27):43782–98. doi: 10.18632/oncotarget.15156 (PMC5546440; doi:10.18632/oncotarget.15156)
Supplement: Supplementary file 1 [file oncotarget-08-43782-s001.pdf]

Recombinant TAT-BMI-1 fusion protein induces ex vivo expansion of human umbilical cord blood-derived hematopoietic stem cells

Supplementary Materials

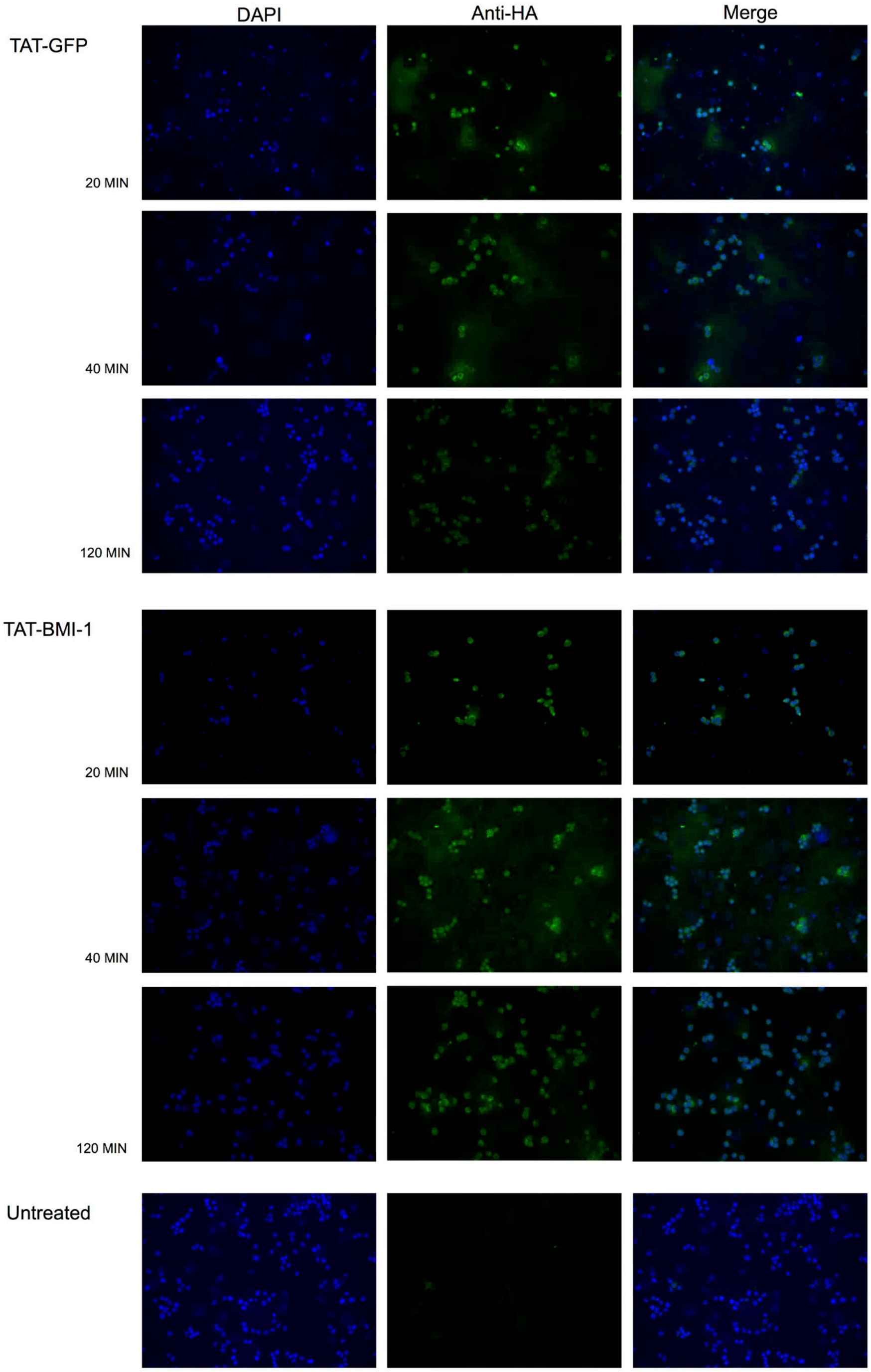

Supplementary Figure 1: Time course of the uptake of TAT-proteins by cord blood derived CD34+ cells. Purified TAT-GFP or TAT-BMI1 proteins were added to cultures of CD34+ cells at a final 100 nM concentration for 20, 40 and 120 min at 37°C. Cytospins were fixed and TAT-proteins were detected by indirect immunofluorescence with rabbit anti-HA antibody and anti-rabbit Alexafluor 488; cell nuclei were counter-stained with DAPI.

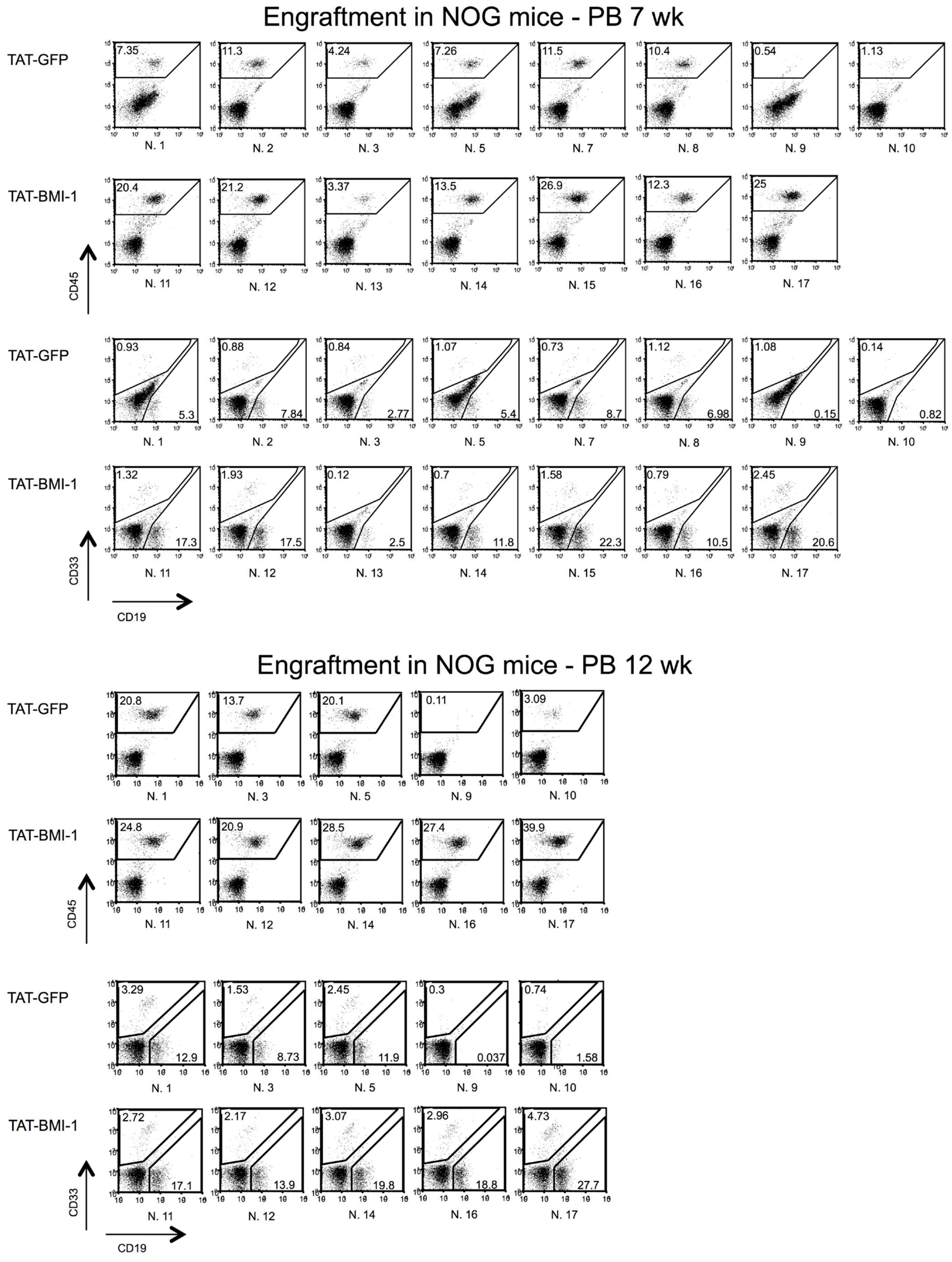

Supplementary Figure 2: TAT-BMI-1 pretreatment of CB-CD34^{+} cells enhances hematopoietic engraftment in NOG mice. Flow-cytometric peripheral blood analysis. Flow cytometric analysis of the peripheral blood of NOG mice transplanted with TAT-GFP or TAT-BMI-1-treated CD34^{+} cells 7 and 12 weeks after injection. Cytograms for each mouse show the percentage of total human leukocytes (CD45^{+}), myeloid (CD33^{+}) and B-lymphoid (CD19^{+}) cells.

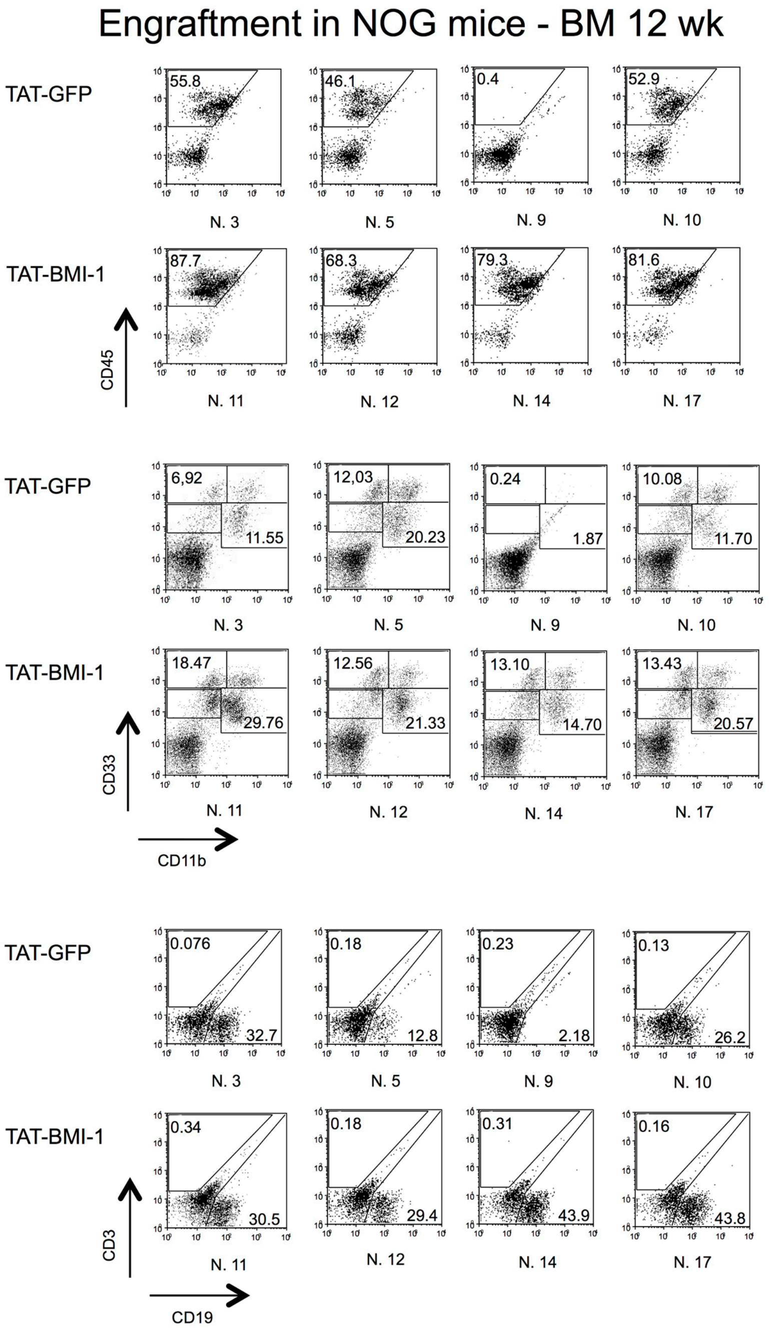

Supplementary Figure 3: TAT-BMI-1 pretreatment of CB-CD34^{+} cells enhances hematopoietic engraftment in NOG mice. Flow-cytometric bone marrow analysis. Flow cytometric analysis of the bone marrow of NOG mice transplanted with TAT-GFP or TAT-BMI-1-treated CD34^{+} cells 12 weeks after injection. Cytograms for each mouse show the percentage of total human leukocytes (CD45^{+}), myeloid (CD11B^{+},CD33^{+}) and lymphoid (CD19^{+}, CD3^{+}) cells.

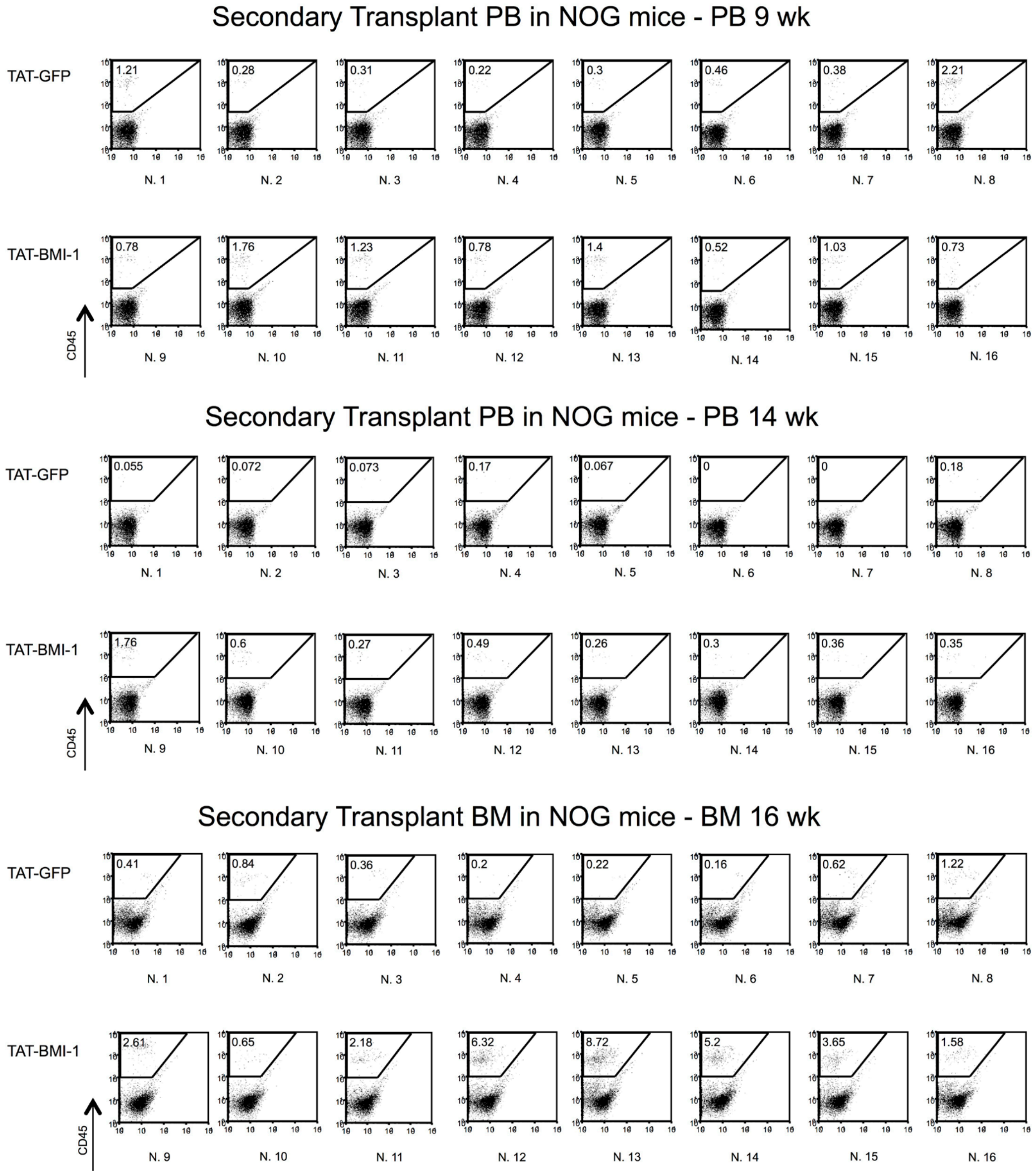

Supplementary Figure 4: TAT-BMI-1 pretreatment of CB-CD34^{+} cells enhances secondary hematopoietic engraftment in NOG mice. Flow cytometric analysis of peripheral blood (weeks 9 and 14 post-transplant) and bone marrow (week 16 post-transplant) of NOG mice transplanted with bone marrow from the primary recipients (shown in Supplementary Figure 3). Cytograms for each mouse show the percentage of total human leukocytes (CD45^{+}).

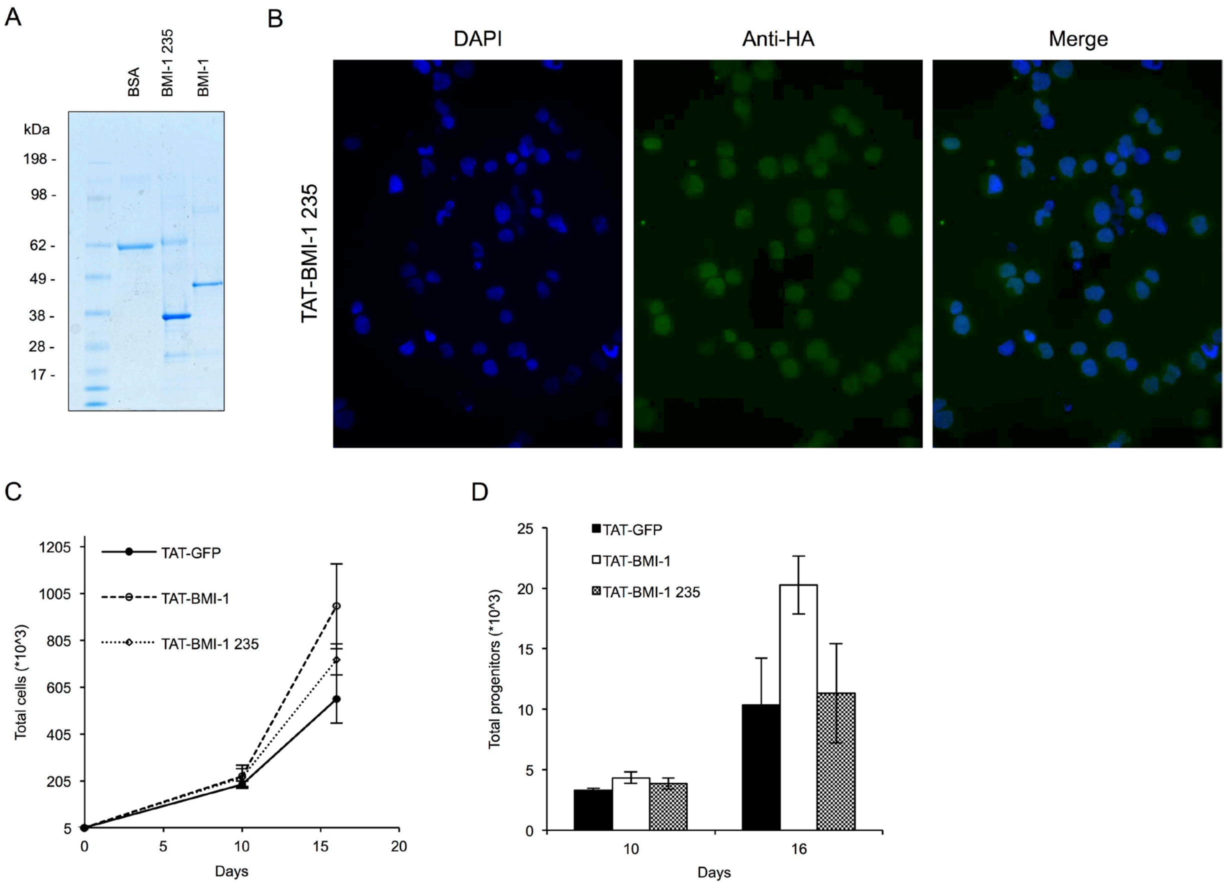

Supplementary Figure 5: Purification and functional characterization of BMI-1 235 on CD34[+] cells expansion. (**A**) Coomassie staining of NUPAGE 4–12% gels of the truncated, 1-235 AA form of TAT-BMI-1 , called TAT-BMI-1 235 (middle lane) 34 kDa, compared with TAT-BMI-1 full length (right lane) 45 kDa and 2 µg Bovine Serum Albumin (BSA) (left lane) 60 kDa. (**B**) Uptake and subcellular localization of TAT-BMI-1 235 in cord blood derived CD34[+] cells. Purified TAT-BMI1 235 was added to cultures of CD34[+] cells at a final 100 nM concentration for 60 min at 37°C. Cytospins were fixed and TAT-BMI1 235 was detected by immunofluorescence with rabbit anti-HA antibody and anti-rabbit Alexa Fluor 488 as described; cell nuclei were counter-stained with DAPI. (40× magnification) (**C**) CD34[+] cells ($5x10^3$/ml) were plated in StemMACS HSC Expansion Medium with StemMACS HSC Expansion Cocktail. During the first 3 days of culture, TAT-BMI-1 235, TAT-BMI-1 and the control TAT-GFP protein were added 4 times a day at a 10 nM concentration. At the time intervals indicated, the cells were counted and re-plated in fresh medium at equal cell densities. Cumulative cell expansion is shown. All assays were performed in triplicate. (**D**) Colony-forming cell (CFC) assays were performed on cells from the cytokine-driven cultures at day 10 and 16×10^3 cells/well were plated in triplicate assays in semisolid StemMACS HSC-CFU medium complete with cytokines. Colonies were scored after 2 weeks and total numbers of progenitors was normalized to the total cell number in the original culture at the time of plating.
